# MicroR-545 enhanced radiosensitivity via suppressing Ku70 expression in Lewis lung carcinoma xenograft model

**DOI:** 10.1186/s12935-015-0207-z

**Published:** 2015-05-28

**Authors:** Chen Liao, Wei Xiao, Nuo Zhu, Zhiyuan Liu, Jiu Yang, Yanhu Wang, Mei Hong

**Affiliations:** Department of Radiotherapy, Nanjing Chest Hospital Affiliated to Southeast University, Guangzhou Road, No215, P.R, Nanjing, Jiangsu 210029 China

**Keywords:** C57BL/6 mice, Ku70, Lewis lung carcinoma cells, miR-545, Radiotherapy

## Abstract

**Objective:**

Radiotherapy is an important therapeutic method for lung cancer. However, in clinical situations, cellular resistance to radiotherapy is a significant component of tumor treatment failure. Thus, clarification in cellular mechanism underlying radiosensitivity of cancer cell is urgently needed. In this study, we established a radiation model of Lewis lung carcinoma in C57BL/6 mice and investigated the possible signaling molecule involved in this process.

**Methods:**

C57BL/6 mice were subcutaneously transplanted with Lewis lung carcinoma cells and locally irradiated followed by measurement in tumor volume. Levels of miR-545 and Ku70 mRNA expression were determined by using Quantitative Real-Time PCR. Expression of Ku70 was determined by using western blot assay. Cell viability was analyzed by MTT assay. Cell apoptosis was examined by using TUNEL assay.

**Results:**

In mice bearing Lewis lung carcinoma tumor, local radiotherapy suppressed tumor growth as well as enhanced expression of miR-545 and downregulated Ku70 level. Inhibition of miR-545 expression reduced radiosensitivity of Lewis tumor. In vitro Lewis lung carcinoma cells experiment, we observed that miR-545 regulated Ku70 expression by targeting Ku70 3′UTR and this process was involved in radiotherapy. This was demonstrated by result of cell proliferation assay in which irradiation reduced apoptosis of cells was mediated by miR-545 inactivation which was reversed by Ku70 silence.

**Conclusion:**

miR-545 increased radiosensitivity of Lewis lung carcinoma via inhibiting Ku70 expression.

## Introduction

Lung cancer continues to be one of the most prevalent malignancies worldwide and is the leading cause of death in both men and women. There is around 20 % of new cancer cases worldwide were diagnosed in China each year; however, more people die from lung cancer than from any other kind of cancer. Importantly, for conveniently be located, approximately 90 % of patients with lung cancer receive external beam radiation treatment as one component of their treatment. However, what is important for radiotherapy is cellular desensitization. Accumulated evidence pointed out that combination of radiation and radiosensitivity-related signaling pathway promotes effectiveness, accuracy and harmlessness of radiotherapy [1, 2]. Thus, exploring the explicit cellular mechanisms of radiotherapy is important.

Ku70 protein, cooperating with Ku80 and DNA-dependent protein kinase (DNA-PK) catalytic subunit (DNA-PKcs), is involved in DNA double-strand break (DNA DSB) repair and V(D)J recombination that is essential for DNA repair, chromosome maintenance, transcription regulation. Recent studies have revealed that Ku70 may be a susceptibility factor for radiation-related cancers. For example, inhibition of Ku70 can increase the radiosensitivity of 786-O cells by enhancing apoptosis [3]. Antisense Ku70 using the antisense nucleic acid strategy increased radiosensitivity of human squamous cell lung carcinoma cell line [4]. Thus, Ku70 might act as a predictor for the effect of radiotherapy in patient with lung cancer [5]. Although there is evidence on increasing ionizing radiosensitivity by Ku70-deficiency in lung cancer, the mechanism regulation on Ku70 expression during radiotherapy is not yet clear.

Previously, expression of Ku70 was regulated by microRNA in brain. MicroRNAs (miRNAs), a class of small noncoding RNAs with approximately 22 nucleotides in length [6, 7] can suppress posttranscriptional gene expression by binding to the 3′-untranslated regions (3′-UTRs) of mRNAs, which induces translational inhibition or target mRNA degradation [8]. miRNAs has been showed to be involved in proliferation [9], differentiation [10], and apoptosis [11] of lung cancer cells, which is linked to tumor formation and progression. Although, it was reported that miRNAs are involved in many signaling pathways and DNA damage repair processes, affecting cellular radiosensitivity [1], to our knowledge, no research has focus on the role of miR-545 in irradiating lung cancer. Previously, miR-545 has been reported to suppress cell proliferation by targeting cyclin D1 and CDK4 in lung cancer cells [12]. Thus, we hypothesized that miR-545 may be involved in the process of radiotherapy in lung cancer.

In this study, we established a radiation model of Lewis lung carcinoma in C57BL/6 mice and aimed to evaluate the role of miR-545 in irradiating tumor. We also directly assessed whether Ku70 was involved in this process and aimed to identify the regulating relationship between miR-545 and Ku70 in the radiotherapy.

## Material and methods

### Mice, cell lines and reagents

Animal experimental protocols were performed in 8–12-week-old female C57BL/6 mice, which obtained from the Institute of Laboratory Animal Science (Chinese academy sciences, Shanghai) and were bred and maintained under specific pathogen-free conditions. All experiment protocols were approved by Medical Experimental Animal Center of Southeast University.

Lewis lung carcinoma cell line was purchased from ATCC (USA) and cultured in RPMI 1640 medium containing 10 % fetal bovine serum. After grew against the wall of flask, formed a monolayer, the cells were digested by 0.25 % trypsin to prepare cell suspensions at 1 × 10^6^/ml.

RPMI 1640 medium and fetal bovine serum were purchased from Gibco (Invitrogen Company, USA). Lipofectamine 2000 transfection reagent was obtained from Invitrogen Life Technologies (Grand Island, NY, USA). Anti-Ku70 antibody was obtained from Santa Cruz Biotechnology Inc. (Santa Cruz, CA, USA).

### Tumor xenograft and irradiation therapy

For tumor transplantation, lewis lung carcinoma cells were transfected with or without miR-545 inhibitor and then suspended in 100 μL PBS and mixed with 20 % matrigel. Tumors were generated by prepared 2 × 10^6^ cells/ml cells subcutaneously injected into the abdomen of C57BL/6 mice. Seven days after the cell inoculated, the mice were divided into two group including control group and radiotherapy group. For radiotherapy, tumors inoculation sites were received a single fraction of 12 Gy/1f/1d. In brief, mice in control group and radiation therapy group were anesthetized by intraperitoneal injection with 10 % chloral hydrate (350 mg/kg). The four limbs were fixed with medical proof fabric. The body surface of tumor location was marked by marker pen and compensated with 1 cm petrolatum gauze. X-ray form 6MV-X (Elekta Limited, West Sussex, UK) vertically irradiated target area under condition of 100 cm Source-skin distance and 12Gy/1f/1d. The irradiated vision is 2.0 cm × 2.0 cm. Notably, mice were kept waking state during radiotherapy. During therapy, the tumor volume was measured using calipers as *length* × *width* × *depth* per two days. Sixteen days after inoculation, animals were euthanized and tumors were removed for Quantitative Real-Time PCR or western blot assay.

### Cell treatment with miRNA inhibitor or mimics

Lewis lung carcinoma cells were treated with miR-545 mimic or miR-545 inhibitor (Ambion Pre-miR miRNA Precursors, Life Technologies) using Oligofectamine (Life Technologies) according to the manufacturer’s instructions. Both miRNA mimics negative control (mimic-NC) and miRNA inhibitor negative control (inhibitor-NC) were severed as negative controls in the experiments respectively. Further analysis of the samples (infection or RNA isolation) was performed at 48 h post-transfection. Lewis lung cancer cells were irradiated after 6 h when pretreated with miR-545-mimics, miR-545-inhibitors, or their negative controls. The sequence for this experiment as follows:

- Hsa-miRNA-545 mimic
- Sense strand 5′-UCAGCAAACAUUUAUUGUGUGC-3′
- Anti-sense strand 5′-GCACACAATAAATGTTTGCTGA-3′
- HSA-miR-545 inhibitor
- 5′-mGmCmAmCmAmCmAmAmUmAmAmAmUmGmUmUmGmCmUmGmA-3′

### MTT proliferation assay

Lewis lung carcinoma cells were seeded at a density of 1400 cells/well. On the next day, cells were irradiated with dose 5 Gy for 24 h. After that, 50 mL of 5 mg/mL 3-(4,5-Dimethylthiazol-2-yl) 22,5-diphenyltetrazolium bromide (MTT) was added to wells for 4 h, and then the media were replaced with DMSO for analysis of optical density using a mQuant Microplate Spectrophotometer (BioTek, UK) at a wavelength of 540 nm.

### Quantitative Real-time PCR

Total RNA was extracted from the Lewis lung carcinoma cells with the Trizol Reagent (Invitrogen, Carlsbad, CA, USA). The mature miR-545 and Ku70 mRNA were quantified by using Quantitative Real-Time PCR (qRT-PCR) assays with fluorescent nucleic acid dye. Each sample (1 μg) was reverse-transcribed into cDNA by using the RealMasterMix First Strand cDNA Synthesis Kit (Tiangen). Real-time PCR was conducted by using SYBR Premix ExTagTM (Takara) according to the manufacturer’s protocols and performed in the Applied Biosystems 7500 Real-time PCR system. The threshold cycle (CT) is defined as the fractional cycle number at which the fluorescence passes the fixed threshold. The miRNA expression levels were normalized to U6 RNA and the Ku70 mRNA levels were normalized to actin mRNA. All reactions were run in triplicate.

### Bioinformatics analysis of miR-545 target in Ku70

Based on bioinformatics analysis, we predicted that hsa-miR-545 can bind with the 3′UTR region of Ku70 by using four common websites (Target Scan: http://www.targetscan.org/, miRanda: http://www.microrna.org/, Microcosm: http://www.ebi.ac.uk/enright-srv/microcosm/cgi-bin/targets/v5/search.pl, and PITA: http://genie.weizmann.ac.il/) (Fig. 3a).

### Plasmid construction and luciferase reporter assays

For Ku70 3′UTR reporter assay, Lewis lung carcinoma cells were placed in 24-well plates (1 × 10^5^ cells per well) and then cotransfected with the pGL3 Luciferase Reporter Vectors (Promega, WI, USA) according to manufactures’ protocol. The mimics and inhibitors of hsa-miR-545 and their negative controls (RIBO Bio, Guangzhou, P.R. China) were cotransfected with the reporter plasmids at a final concentration of 100 nmol/μl. 48 h after transfection in Lewis lung cancer cells, luciferase activity in lysates was measured with a Dual-Luciferase Reporter Assay System (Promega, WI, USA) followed by the manufacture’s suggestions and normalized against the activity of the pRL-SV40. Independent triplicate experiments were performed for each plasmid construct.

### TUNEL staining

The TUNEL reaction which based on labeling of DNA strand breaks was conducted to detect cells apoptosis and performed with an in situ cell detection kit (Roche Applied Science, Indianapolis, IN, USA), according to the manufacturer’s instruction. In brief, sections in 2 changes of xylene for 5 min each, and hydrate with 2 changes of 100 % ethanol for 3 min each, and 95 % ethanol for 1 min followed by rinsing in distilled water. After pretreated with proteinase K, cells were cultured in TdT Reaction Buffer for 10 min and then conducted TdT Reaction in TdT Reaction Mixture for 1–2 h at 37–40 °C in humidified chamber. The reaction was stopped with washing buffer and the sample was resuspended in PBS containing FITC-Avidin D for 30 min at room temperature. The reaction mixture was counterstained with PI or DAPI for 20 min. TUNEL-positive nuclei (stained brown) were counted by Motic Images Advanced 3.2 in 10 random fields (×200), and then averaged.

### Western blot

Proteins in Cells were extracted by inM-PER mammalian protein extraction reagent (Pierce Biotechnology) followed by centrifugation at 15 000 g for 10 min. Protein concentration of cell lysates was measured by using DC protein assay kit (Bio-Rad). Proteins (10–20 mg) were separated by 10 % SDS-polyacrylamide gel electrophoresis and transferred to PVDF membrane from Bio-Rad (Hercules, CA, USA). Two hours after blocking in 5 % nonfat milk, the protein blots were then incubated with primary antibodies in 3 % bovine serum albumin at 4 °C overnight, followed by incubation with secondary antibodies at room temperature for 2 h. The protein signals were detected by ECL method.

### Ku70 interference

Lewis lung carcinoma cells were treated with Ku70 siRNA or scrambled siRNA as negative control according to the manufacturer’s instructions. Briefly, siRNA and OligofectAmine were mixed separately with OptiMem and incubated for 15 min at room temperature. And then, these reagents were combined and incubated for 15 min before adding to the cells in RPMI 1640 without penicillin and streptomycin. Notably, Ku70 siRNA was cotransfected with miR-545 inhibitor. Twenty-four hours after transfection, the cells were irradiated with a single dose of 5 Gy/1f/1d for 24 h. And then the cell viability and apoptosis were examined accordingly.

### Statistical analysis

All statistical analyses were performed using SPSS 16.0 software (Chicago, IL). Student’s *t* test was used to analyze the significance between two groups. Error bars represent SD. *P*-Values < 0.05 were considered statistically significant.

## Results

### Local radiotherapy suppressed tumor growth in mice bearing Lewis lung carcinoma tumor

**Fig. 1:**
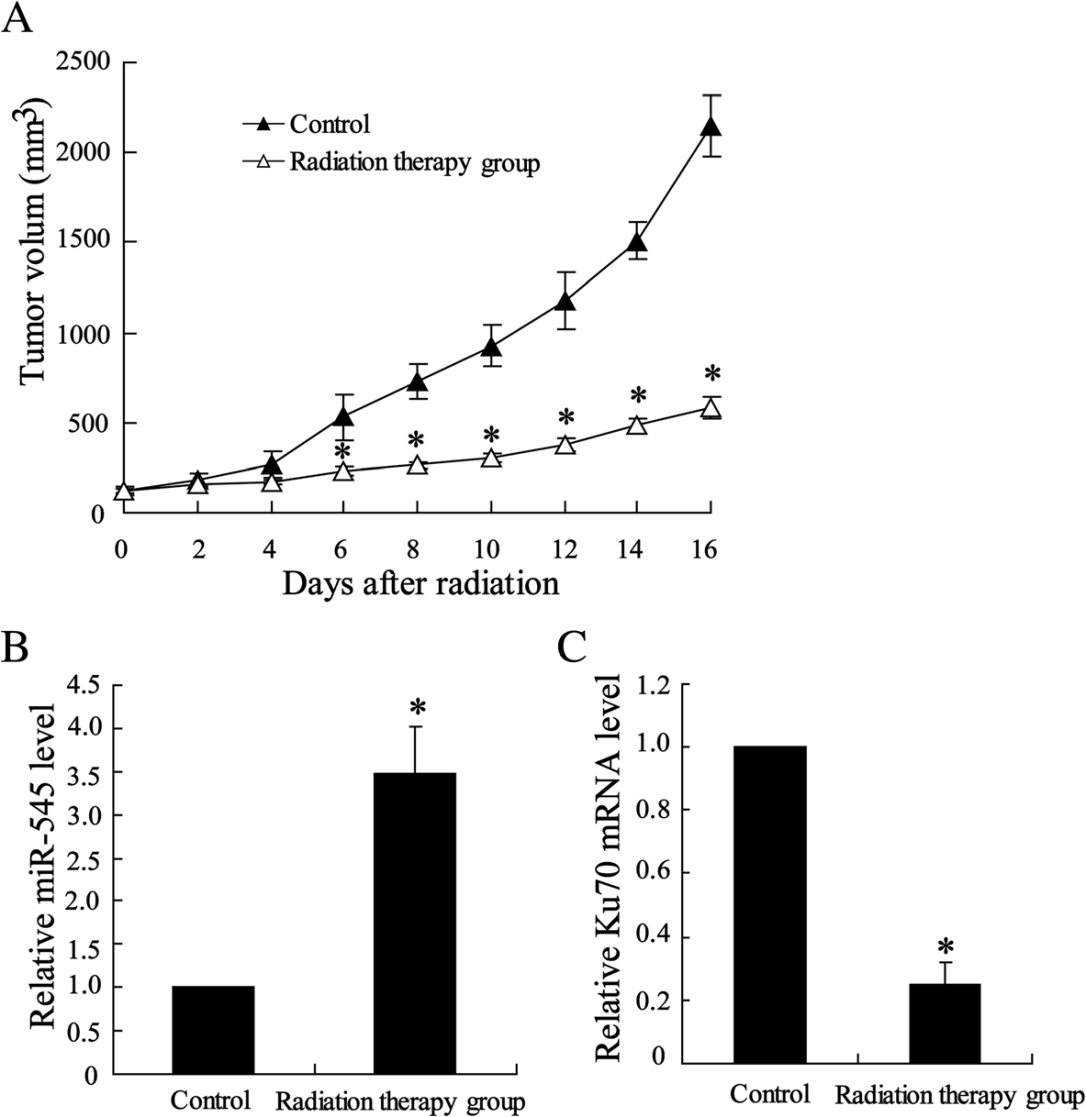
Effect of local high-dose irradiation on tumor growth and expression of miR-545 and Ku70 in mice bearing Lewis lung carcinoma tumor. Seven days after C57BL/6 mice transfected with Lewis lung cancer cell lines, tumors were irradiated with a single dose of 12 Gy/1f/1d, whereas control mice received no radiotherapy. (**a**) Growth of tumor in irradiated mice. (**b**) Expressions of miR-545 and (**c**) Ku70 were determined by using Quantitative Real-Time PCR in tumors. The results were represented as mean ± SD. ^*^ *P* < 0.05 compared with control

After subcutaneous transplantation, C57BL/6 mice bearing Lewis lung carcinoma tumors were locally irradiated with a single dose of 12 Gy for 7 days, which resulted in a significant reduction in tumor growth (Fig. 1a). Upon to qRT-PCR analysis on expression of miR-545 and Ku70 in radiation therapy tumor, the results showed an increase in miR-545 level (Fig. 1b) and a decline in Ku70 level mRNA compared with untreated tumors (Fig. 1c).

### Suppression of miR-545 expression reduced radiosensitivity of tumor in mice bearing Lewis lung carcinoma tumor

**Fig. 2:**
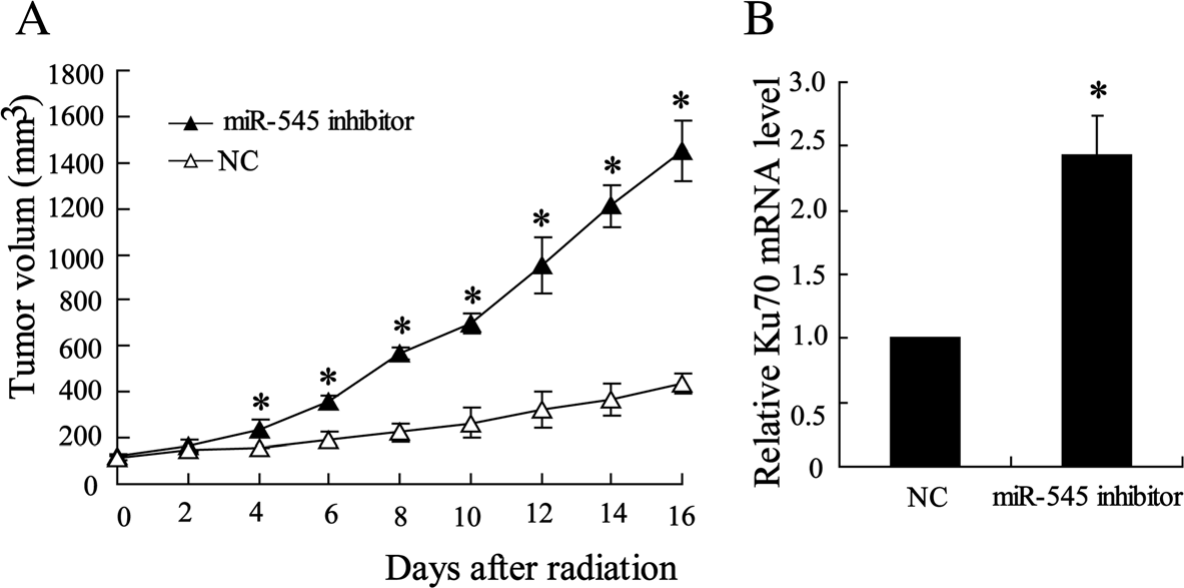
Effect of silenced miR-545 on radiosensitivity of tumor and Ku70 miRNA expression. Before injected into C57BL/6 mice, the Lewis lung cancer cells were transfected with miR-545 inhibitor. Tumors were irradiated with a single dose of 12 Gy/1f/1d as in Fig. 1. (**a**) Growth of tumor in irradiated mice. (**b**) Expression of Ku70 was determined by using quantity RT-PCR in tumors. The results were represented as mean ± SD. ^*^ *P* < 0.05 compared with negative control

To determine the effect of miR-545 on the modulation of radiotherapy of tumors, Lewis lung carcinoma cells were transfected with miR-545 inhibitor before subcutaneous transplantation in C57BL/6 mice. After locally irradiated with a single dose of 12 Gy/1f/1d, the tumors volume was much bigger than that of untreated tumors during irradiation (Fig. 2a). Additionally, compared with negative control, knock down of miR-545 expression upregulated Ku70 expression in tumor (Fig. 2b).

### Regulated Ku70 expression by miR-545 in Ku70 3′UTR

**Fig. 3:**
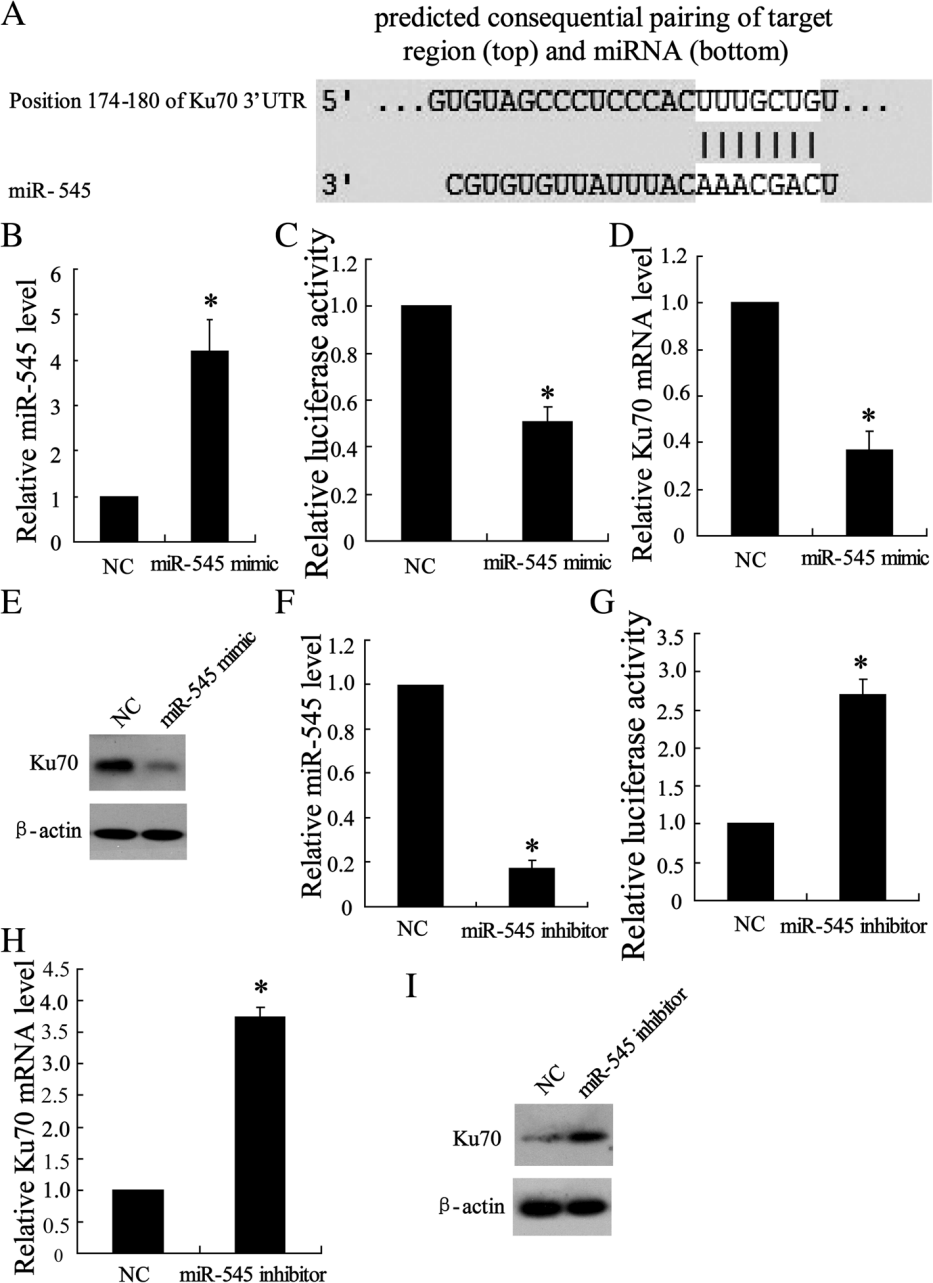
Targeting site of miR-545 in the Ku70 3′UTR. **a** Ku70 3′UTR was predicted a binding site for hsa-miR-545 and mRNA sequence is shown with potential binding sites indicated in white. The highly conserved mature miR-545 sequence in mammals and potential binding between the miR-545 seed region to the mouse Ku70 3′UTR sequence are shown. 24 h after transfected with miR-454 mimic, Lewis lung carcinoma cells were lyzed to examine (**b**) miR-454 expression by using quantity RT-PCR, (**c**) Ku70 3′UTR activity using luciferase report, (**d**) Ku 70 mRNA level by using quantity RT-PCR and (**e**) Ku 70 protein expression by using western blot assay. 24 h after transfected with miR-454 inhibitor, Lewis lung cancer cells were lyzed to examine (**f**) miR-454 level, (**g**) Ku70 3′UTR activity, (**h**) Ku 70 mRNA level and (**i**) Ku 70 protein expression by using the same detection as before. The results were represented as mean ± SD. ^*^ *P* < 0.05 compared with negative control

Ku70 was regarded as a potential target gene of miR-545 using TargetScan Release 5.2 in which we found a binding site for miR-545 in the 3′UTR of Ku70 mRNA (Fig. 3a). To confirm the key regulated role of miR-545 in Ku70 expression in tumors, Lewis lung carcinoma cells were transfected with miR-545 mimic to overexpression miR-545 which was shown in Fig. 3b. The entire 3′UTR of Ku70 was inserted downstream of the luciferase gene and assayed in Lewis lung carcinoma cells and the result showed a decrease (45 %) in luciferase activity (Fig. 3c). Upon to expression of Ku70, transfected miR-545 mimic downregulated both Ku70 mRNA (Fig. 3d) and Ku70 protein expression (Fig. 3e). Additionally, Lewis lung carcinoma cells were transfected with miR-545 inhibitor to silence miR-545 expression which was shown in Fig. 3f. The data of luciferase reporter gene assay showed that knock down miR-545 leaded to increase of Ku70 transcriptional activity (Fig. 3g). In the case of expression of Ku70, transfected with miR-545 inhibitor upregulated both Ku70 mRNA level (Fig. 3h) and Ku70 expression (Fig. 3i).

### Regulated Ku70 expression by miR-545 in irradiated-Lewis lung carcinoma cells

**Fig. 4:**
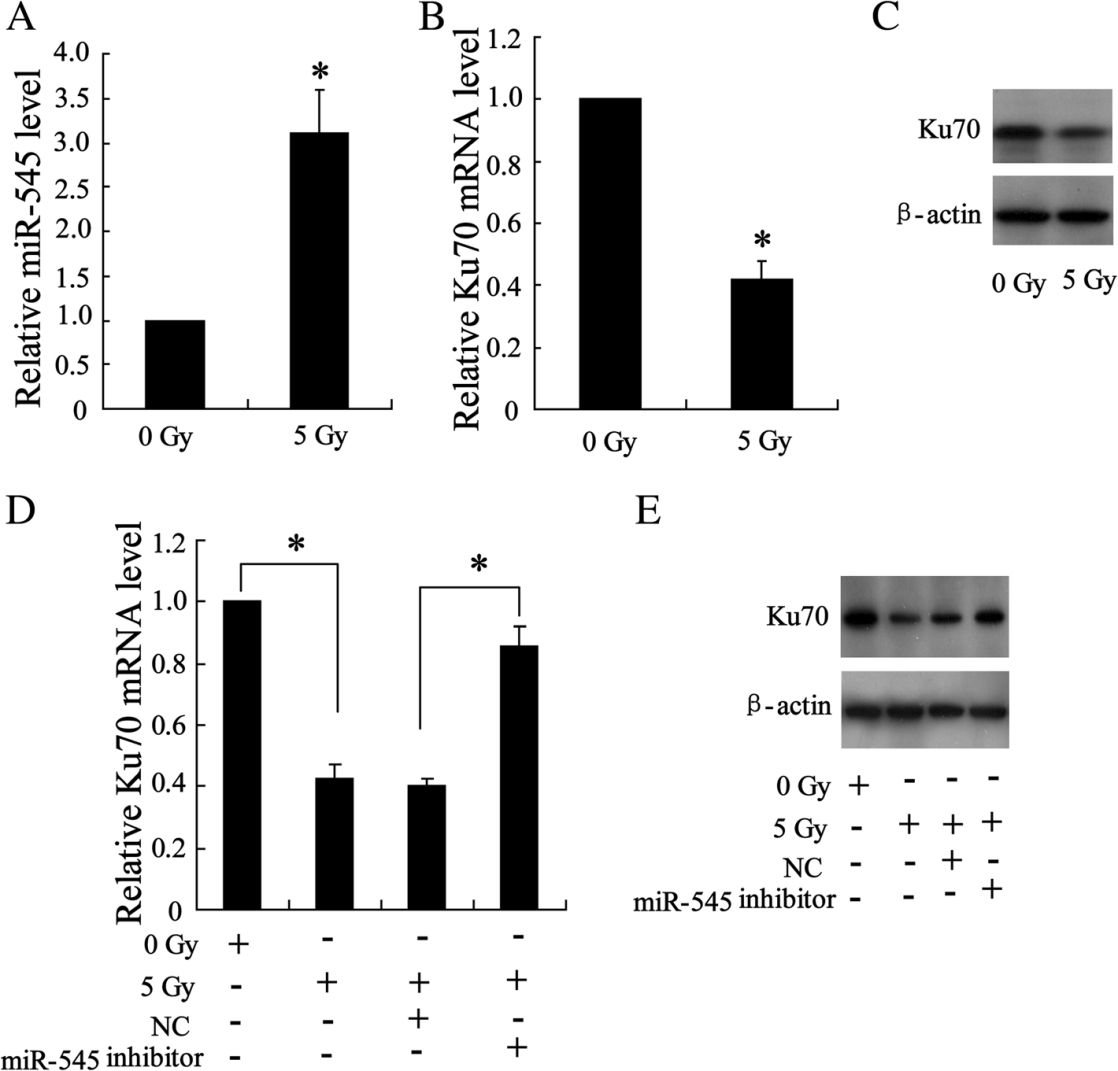
Effect of high-dose irradiation on expression of miR-545 and Ku70 in Lewis lung cancer cells. 24 h after irradiated with a single dose of 5 Gy/1f/1d, cells were lyzed to detect expression of miR-545 (**a**) and Ku70 mRNA by using quantity RT-PCR (**b**) and Ku protein by western blot (**c**). The cells were transfected with miR-545 inhibitor and then exposed to radiation followed by detection on expression of Ku70 mRNA (**d**) and Ku70 protein (**e**). The results were represented as mean ± SD. *P < 0.05 compared with corresponding control

As indicated in Fig. 3, miR-545 exerted negative impact on expression of Ku70 in Lewis lung carcinoma cells. To ascertain this regulational process occurred during radiotherapy, Lewis lung carcinoma cells were irradiated with a single dose of 5 Gy/1f/1d. Consequently, irradiation increased expression of miR-545 (Fig. 4a), but suppressed expression of Ku70 in levels of Ku70 mRNA (Fig. 4b) and Ku70 protein (Fig. 4c). Furthermore, miR-545 was silenced by cells being transfected with miR-545 inhibitor. Reversely, expression of Ku70 inhibited by irradiation was upregulated in levels of Ku70 mRNA (Fig. 4d) and Ku70 protein (Fig. 4e).

### Interaction between miR-545 and Ku70 in irradiation-induced cells apoptosis

**Fig. 5:**
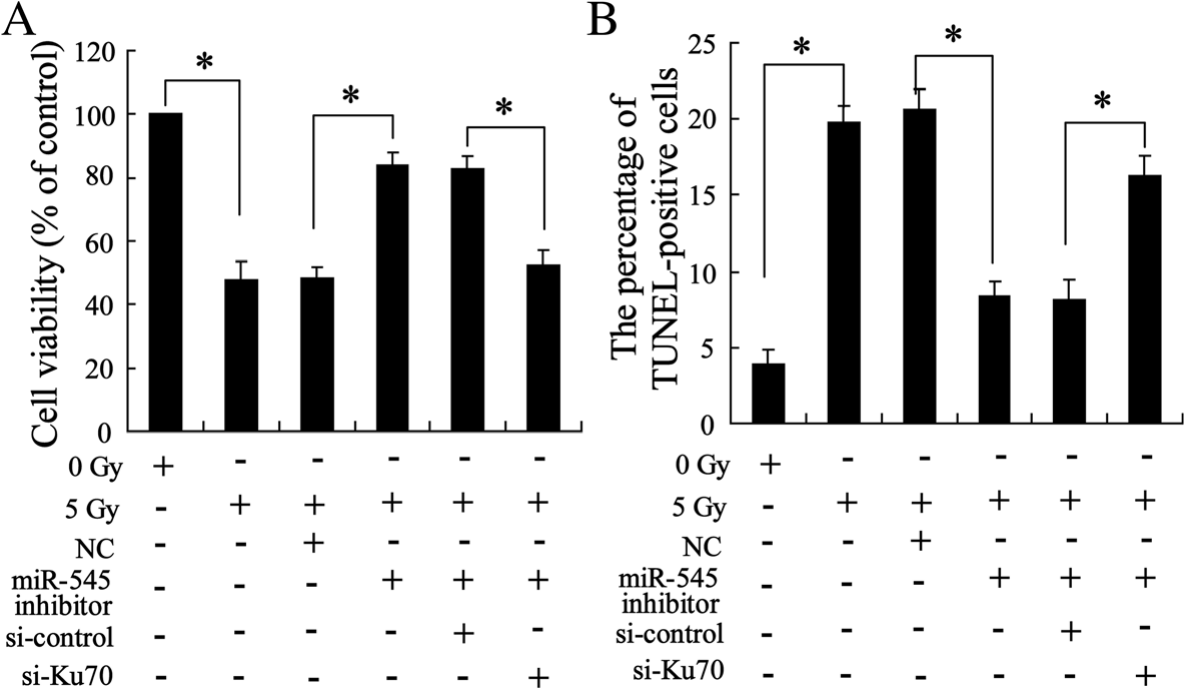
Effect of irradiation on cell proliferation. The cells were transfected with miR-545 inhibitor or si-Ku70 and then irradiated with a single dose of 5 Gy/1f/1d for 24 h. (**a**) cell viability by using MTT assay. (**b**) Cell apoptosis by using TUNEL assay

The above results indicated that the regulation of Ku70 expression by miR-545 is involved in the process of radiotherapy of Lewis lung cancer. Next, we tried to detect whether this process affects Lewis lung cancer cells proliferation. As shown in Fig. 5a, cell viability was reduced by irradiation and was increased by cell pretransfected with miR-545 inhibitor. However, this increase was reversed by cells cotransfected with si-Ku70 and miR-545 inhibitor suggesting the essential signaling of inhibition of Ku70 expression by miR-545 in radiotherapy. Furthermore, regulated Ku70 expression by miR-545 was also involved in irradiation-induced apoptosis of Lewis lung carcinoma cells (Fig. 5b).

## Discussion

Reduction of radiation susceptibility commonly resulted in failure of cancer therapy. Thus much attention has been focused on the identification the detailed mechanism underlying radiotherapy to obtain the possible therapy target. In the present study, we established X ray irradiated in vitro rat model of xenografted lung tumor and in vivo Lewis lung Carcinoma cells. In results, we observed that radiotherapy enhanced miR-545 expression while inhibited Ku70 expression in both irradiated xenografted tumor and cells. Moreover, our data also showed that upregulated miR-545 suppressed Ku70 transcriptional activity by targeting its 3′UTR, which plays a vital role in radiotherapeutic sensitivity.

It is well accepted that miRNAs were the key regulator for various cellular functions [13]. Accumulated evidence pointed out that miRNAs are also involved in many signaling pathways and DNA damage repair processes, which is essential for cancer radiotherapy [1]. In this present research, we confirmed and are the first to note that the miR-545 was upregulated during irradiation of Lewis lung carcinoma tumor in vivo and in vitro. Based to its inhibition effect on proliferation of lung cancer cell [12], overpression of miR-545 was assumed to exert resistance impact on tumor growth during radiotherapy, which was supported by tumor growth inhibition in vivo and Lewis cell apoptosis in vitro in our research. Notably, inhibition of miR-545 desensitized radiation of tumor. Given this, miR-545 might be a therapy site for high irradiation efficiency.

In consideration of high human lung cancer causes many deaths worldwide, elucidation on the molecular mechanism underlying the radioresistance lung cancer cells is particularly important. DNA double-strand breaks (DSBs) caused by ionizing radiation leads to different kind of DNA damages that can result in tumor growth inhibition in cancer cells [14]. The nonhomologous end joining (NHEJ) pathway, however, is regarded as the major pathway for the repair of radiation induced DSBs in mammalian cells [15, 16]. One of the main participants in this pathway is the DNA-dependent protein kinase (DNA-PK) that consists of a large catalytic subunit, DNA-PKcs, and a heterodimeric protein named Ku, which is a highly stable protein complex consisting of Ku70 and Ku80 subunits [17, 18]. Ku is the initiated protein for DSBs repair and it recognizes DSBs and recruits additional pathway components to process and repair the damage. In this study, Ku70 founction as an indicator for DSBs repair. Although it was reported that Ku70 expression was regulated by miRNA in brain [12], there is no evidence on regulation of its synthesis by miR-545 in lung cancer. We confirmed the possible targeting region of Ku70 mRNA by miR-545 in 3′UTR by using bioinformatics analysis. Moreover, it was supported by that inhibition of miR-545 increased Ku70 3′UTR activity and Ku70 protein expression.

In conclusion, we established Lewis Lung Carcinoma bearing mice in C57BL/6 mice. The findings suggest that miR-545 plays an important role in the mechanism underlying the radioresistance of Lewis Lung Carcinoma in vivo and in vitro. Further studies to elucidate the molecular mechanism underlying the radioresistance of the Lewis Lung Carcinoma cell lines indicated that over-expressed miR545 inhibited Ku70 activity. These founding will contribute to a better understanding of not only the molecular mechanisms of radiotherapy in lung epithelial cells, but also the development of combination between new gene therapies and radiotherapies for types of malignancies.
